# Problematic Social Media Use in Adolescents and Young Adults: Systematic Review and Meta-analysis

**DOI:** 10.2196/33450

**Published:** 2022-04-14

**Authors:** Holly Shannon, Katie Bush, Paul J Villeneuve, Kim GC Hellemans, Synthia Guimond

**Affiliations:** 1 Department of Psychiatry The Royal's Institute of Mental Health Research University of Ottawa Ottawa, ON Canada; 2 Department of Neuroscience Carleton University Ottawa, ON Canada; 3 School of Mathematics and Statistics Carleton University Ottawa, ON Canada; 4 Department of Psychoeducation and Psychology Université du Québec en Outaouais Gatineau, QC Canada

**Keywords:** problematic social media use, depression, anxiety, stress

## Abstract

**Background:**

Technology is ever evolving, with more and more diverse activities becoming possible on screen-based devices. However, participating in a heavy screen-based lifestyle may come at a cost. Our hypothesis was that problematic social media use increased the prevalence of mental health outcomes.

**Objective:**

This study seeks to systematically examine problematic social media use in youth and its association with symptoms of depression, anxiety, and stress.

**Methods:**

A systematic search was conducted to identify studies in adolescents and young adults, using the databases Engineering Village, Psycinfo, Pubmed, and Web of Science. A total of 18 studies were identified, with a total of 9269 participants in our review and included in the meta-analysis.

**Results:**

Our metaregression shows moderate but statistically significant correlations between problematic social media use and depression (*r*=0.273, *P*<.001), anxiety (*r*=0.348, *P*<.001), and stress (*r*=0.313, *P*<.001). We did not find evidence of heterogeneity of these summary correlations by age, gender, or year of publication.

**Conclusions:**

This study provides further evidence of the association between problematic social media use and negative mental health among adolescents and young adults and supports future research to focus on the underlying mechanisms of problematic use of social media.

**Trial Registration:**

PROSPERO CRD42021222309; https://tinyurl.com/2p9y4bjx

## Introduction

Technology is ever evolving, with more and more diverse activities becoming possible on screen-based devices. With this increasing engagement in the digital world, social networking sites have become an increasingly popular activity, especially among younger populations [1]. Adolescents and young adults represent a unique population in terms of social media users, as they are the first generations to grow up in a highly digitized society. Social media use is highly normative among young individuals: In 2016, 97.5% of young adults in the United States reported using at least one social media site regularly [2]. However, participating in a heavy screen-based lifestyle may come at a cost. A wealth of evidence suggests higher levels of social media use are associated with symptoms of anxiety [3-5], symptoms of depression [3,6-8], decreased psychological well-being [9], lower self-esteem [3], psychological distress [10-12], and loneliness [5]. A meta-analysis in young adults reports a small correlation between depressive symptoms and adolescent social media use, defined by frequency of use [13]. However, along with the evidence supporting the negative impacts of social media use, some reports suggest there may exist positive outcomes following use. For example, social media use has also been linked to higher quality of life, social support, well-being, and reduced stress [14,15].

Aside from excessive use of social media, typically defined on the basis of hours of use, the term of problematic use characterizes individuals who experience addiction-like symptoms as a result of their social media use [5]. Problematic social media use reflects a non–substance related disorder by which detrimental effects occur as a result of preoccupation and compulsion to excessively engage in social media platforms despite negative consequences [16]. While there exists no official diagnostic term or measurement, Andreassen et al [17] developed the Facebook Addiction Scale, which measures features of substance use disorder such as salience, tolerance, preoccupation, impaired role performance, loss of control, and withdrawal, to systematically score problematic Facebook use. This scale has been widely used to conceptualize problematic use as a behavioral addiction and has therefore also been modified to measure overall problematic social media use, instead of focusing on Facebook specifically [18]. Similar to high frequencies of social media use, problematic social media use has also been associated with poor mental health outcomes such as depression, anxiety, decreased well-being, and lower self-esteem [1,17,19-22]. A recent meta-analysis by Cunningham et al [23] found that problematic social media use was a stronger predictor of depressive symptoms when compared to the measure of time spent on social networking sites. Therefore, based on previous evidence, problematic social media use may be more imperative to examine than hours spent on social media platforms.

Researchers recognize youth and students as a vulnerable group compared to adults because their increased use of social media is occurring during a time of identity formation, where they are free to explore various life possibilities and develop new values [2]. Furthermore, their use occurs when critical brain circuits involved in emotion regulation and motivation are continuing to undergo development [24]. As social media plays a large role in their day-to-day lives, patterns and frequency of use have the potential to become problematic. On this level, youth are more at risk for facing cyberbullying [25], finding it difficult to disengage from the media and allowing it to interfere with their social relationships [26]; this in turn puts them at risk for experiencing negative emotional and psychosocial outcomes [27]. Therefore, younger individuals are a vulnerable group of social media users, and it is important to better understand the outcomes for well-being that are associated with this type of problematic social media use. Yet, the magnitude of impact social media has on adolescents and emerging adults, especially when considering problematic use, remains unclear.

With this background, we systematically examined and summarized, with the most current evidence, the strength of association between problematic social media use and multiple mental health outcomes. Specifically, we considered depressive symptoms, anxiety symptoms, and stress. Our a priori hypothesis was that problematic social media use adversely impacts all mental health outcomes measured. In addition, age, gender, and year of publication were investigated as covariates in the relationship between problematic social media use and all mental health outcome variables.

## Methods

This meta-analysis was registered with the International Prospective Register of Systematic Reviews (PROSPERO; CRD42021222309). The PRISMA (Preferred Reporting Items for Systematic Reviews and Meta-Analyses) guidelines were followed [28].

### Inclusion and Exclusion Criteria

This systematic review included measures of problematic social media use, with depressive symptoms, anxiety symptoms, and stress as outcome measures, assessed by validated instruments. The studies included were cross-sectional and provided a measure of association between problematic use and at least one of the mental health outcomes. Studies must have included a measure of problematic use from the participants; simply indicating if the participant was a user of social media was not acceptable (eg, grouping users vs nonusers of social media). Social media use was also examined in general, without focusing on specific activities (eg, studies looking at specific screen content or comparisons on social media platforms, etc) or a specific platform (eg, Facebook). Problematic social media use scales must have been validated to specifically measure social media use in terms of addictive use, comprising criteria used when measuring substance use disorders. Studies included were restricted to English language, and ages 12 to 30 years. Studies were excluded if they only measured frequency or problematic use of the internet in general, as social media use specifically must have been measured. Studies were also excluded if social media was being used as a treatment/intervention or in a focus-group setting. Finally, studies were excluded if they only measured social media use in clinical populations.

### Literature Search

A systematic literature search was conducted in April 2021 using the databases Engineering Village, PsycInfo, Pubmed, and Web of Science using the terms “social media,” “social networking,” “mental health,” “depression,” “depressive symptoms,” “anxiety,” and “stress.” These search terms were used to quantify social media use in terms of problematic use.

### Assessment of Quality

All eligible studies were assessed for quality using an adapted version of the Newcastle-Ottawa quality assessment scale for cross-sectional studies, which was used to score the risk of bias for each study [29]. All studies were independently rated by HS and KB and given a score out of 10. Conflicts in scoring were resolved by discussion (Multimedia Appendix 1).

### Data Extraction

For each study identified as eligible, the following information was extracted; study identification (authors, year of publication, and country conducted), study design (sample size, age range, mean age, gender, and questionnaire used to measure problematic social media use), outcome variables (questionnaire used to measure each outcome and measure of association). See Multimedia Appendix 1 for questionnaires used to measure problematic use and outcome variables for each study included in the meta-analysis.

### Statistical Analysis

To quantify the association between problematic social media use and depressive symptoms, anxiety symptoms, and stress, we used the Pearson correlation coefficient (*r*). Problematic use was considered on a continuum, based on the score obtained from the questionnaire used, which measures problematic use as addiction-like tendencies. All data analysis was performed using the statistical software Stata (Stat Corp) [30]. A random effects model was used, as it does not assume a common effect size across studies. The variance of *r* was calculated in order to obtain the standard error for each correlation coefficient. The effect size in all groups of analysis had a 95% confidence interval. Publication bias was evaluated by producing a funnel plot, and by performing the Egger test. Age, gender, and year of publication were investigated as covariates by adding mean age, the percentage of male participants reported, and publication year for each study into separate metaregression analyses.

### Ethical Considerations

Since meta-analyses do not need Institutional Review Board approval, the authors did not seek ethics approval.

## Results

### Literature Search

**Figure 1:**
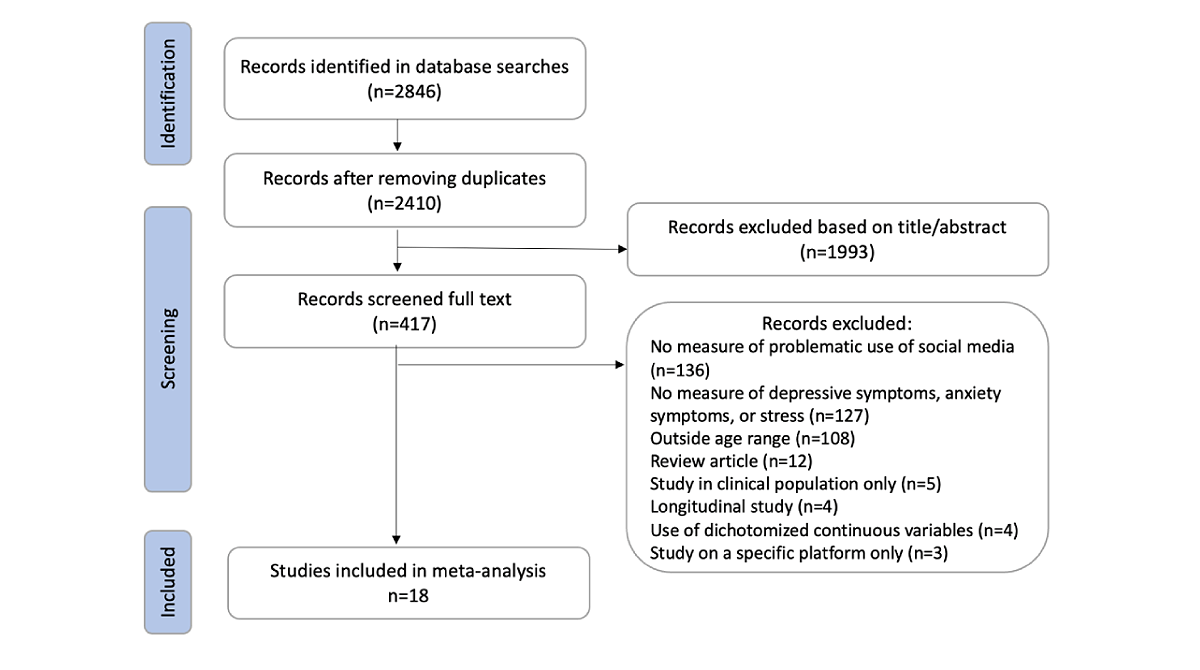
Flow chart of the search process and studies included.

The literature search yielded 2846 articles, with 2410 articles remaining after duplicates were removed (Figure 1). Articles were screened based on titles and abstract to remove any records that were not quantitative, did not assess one of the outcomes, or were longitudinal. After the first screening, 417 (17.30%) articles were considered to be eligible and were then screened based on full text to exclude any remaining records that did not meet the inclusion criteria. Of the remaining articles, 4 were excluded as they were reporting results using dichotomized continuous variables. These studies separated participants into groups based on the scores of their respective scales, and therefore could not be used in our meta-analysis. Additionally, any unpublished data were obtained by contacting the corresponding author. One study included reported statistics distinct to two separate samples; therefore, the two samples were coded independently [31]. The results from Kim et al [32] were excluded from the metaregression, as mean age was not reported or received when contacted. The correlation from Giordano et al [33] with problematic social media use was reported as a combined score of depressive and anxiety symptoms, which therefore could not be included in the meta-analysis. However, all variables were pooled together for the metaregression analyses, so they were included when examining age, gender, and publication year as covariates. Details on the final 18 studies and 9269 total participants included are summarized in Table 1.

**Table 1:** Summary of included studies on the relationship between social media use and outcome variables (note that not all studies measured all three outcomes. Giordano et al [33] assessed anxiety and depression combined and was therefore only included in the meta-regression analyses).

First author (year)	Sample size	Female, n (%)	Male, n (%)	Age (years) range (mean)	Country	Problematic use and depression (*r*)	Problematic use and anxiety (*r*)	Problematic use and stress (*r*)	Problematic use and depression and anxiety combined (*r*)
Holmgren (2017) [34]	442	228 (51.6)	214 (48.4)	18-21 (18.86)	United States	0.29	N/A^a^	N/A	N/A
Wang (2018) [35]	365	190 (52)	175 (48)	14-18 (16.29)	China	0.18	N/A	N/A	N/A
Apaolaza (2019) [36]	346	179 (51.7)	167 (48.3)	17-26 (18.73)	Spain	N/A	N/A	0.49	N/A
Hou (2019) [37]	641	477 (74.4)	164 (25.6)	17-25 (19.9)	China	0.22	0.22	0.11	N/A
Kircaburun (2019) [25]	470	280 (59.6)	190 (40.4)	14-18 (16.29)	Turkey	0.03	N/A	N/A	N/A
Mitra (2019) [38]	264	164 (62.2)	100 (37.8)	18-25 (21.56)	India	0.39	N/A	N/A	N/A
Chen (2020) [39]	437	308 (70.5)	129 (29.5)	16-30 (24.21)	China	N/A	0.29	N/A	N/A
Kim (2020) [32]	209	31 (14.8)	178 (85.2)	15-18 (N/A)	China	N/A	0.20	N/A	N/A
Kircaburun, Demetrovics (2020) [40]	344	282 (82)	62 (18)	18-25 (20.87)	Turkey	0.22	N/A	N/A	N/A
Kircaburun, Grifiths (2020) [41]	460	281 (61)	179 (39)	18-26 (19.74)	Turkey	0.34	N/A	N/A	N/A
Stockdale (2020) [26]	385	204 (53)	181 (47)	17-19 (18.01)	United States	0.28	0.24	N/A	N/A
Wong (2020) [42]	300	178 (59.3)	122 (40.7)	18-24 (20.89)	Hong Kong	0.336	0.344	0.384	N/A
Yildiz (2020) [43]	451	214 (47.5)	237 (52.5)	13-17 (15.5)	Turkey	N/A	0.58	N/A	N/A
Brailovskaia; Lithuanian sample (2021) [31]	1640	1123 (68.5)	517 (31.5)	18-29 (19.09)	Lithuania	0.305	0.329	0.246	N/A
Brailovskaia; German sample (2021) [31]	727	548 (75.4)	179 (24.6)	18-29 (21.47)	Germany	0.396	0.461	0.411	N/A
Giordano (2021) [33]	428	218 (50.9)	210 (49.1)	13-19 (17.38)	United States	N/A	N/A	N/A	0.314
He (2021) [44]	218	218 (100)	0 (0)	19-23 (19.6)	China	N/A	N/A	0.23	N/A
Kilincel (2021) [45]	1142	722 (63.2)	420 (36.8)	12-18 (15.6)	Turkey	N/A	0.417	N/A	N/A

^a^N/A: not applicable.

### Problematic Social Media Use and Depressive Symptoms

**Figure 2:**
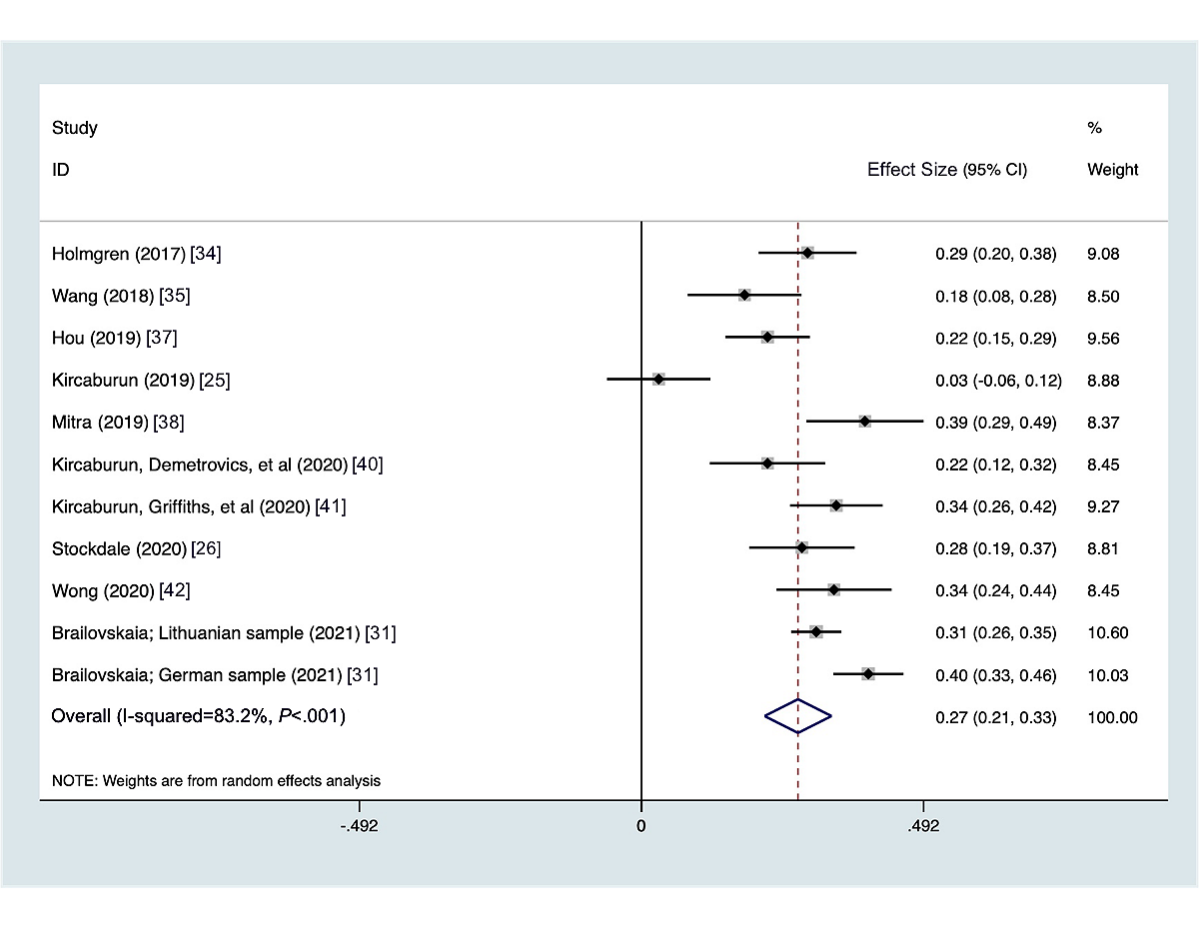
Forest plot of depressive symptoms and problematic social media use by year.

When examining depression as an outcome, 11 studies presented associations between problematic social media use in adolescents and young adults. The Center of Epidemiologic Studies Depression Scale was most commonly used to measure depressive symptoms. The summary metaregression correlation between problematic social media use and depressive symptoms was 0.273 (95% CI 0.215-0.332, *P*<.001). There was heterogeneity in the measures of association across the studies (Figure 2) with an I^2^=83.2%, Q^2^=59.69, and *P*<.001. The funnel plot (Multimedia Appendix 1) shows slight asymmetry, suggesting slight publication bias, however Egger’s test for small-study effects was not significant (*P*=.35).

### Problematic Social Media Use and Anxiety Symptoms

**Figure 3:**
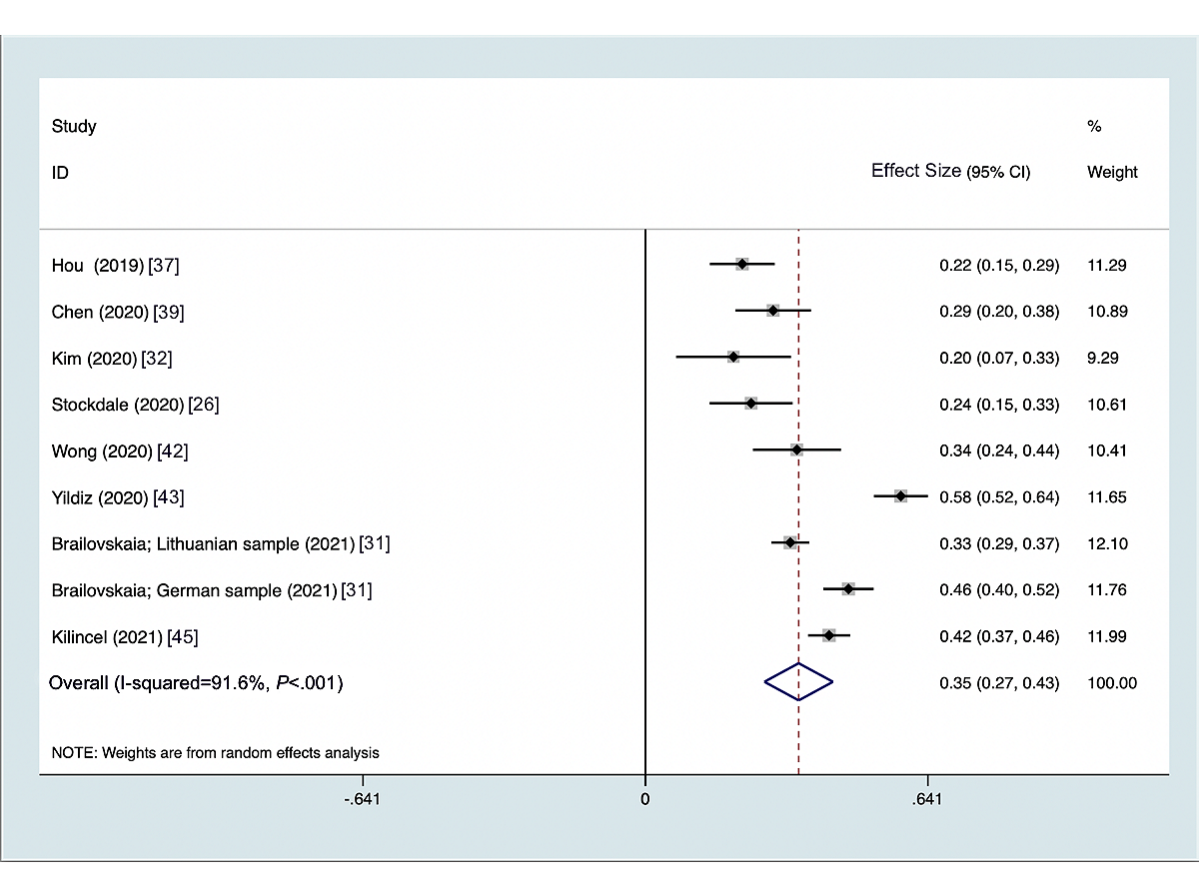
Forest plot of anxiety symptoms and problematic social media use by year.

When examining anxiety symptoms as an outcome, 9 studies were identified measuring an association with problematic social media use in adolescents and young adults. The Depression Anxiety Stress Scale was most commonly used to measure anxiety symptoms. The summary metaregression correlation between problematic social media use and anxiety symptoms was 0.348 (95% CI 0.270-0.426, *P*<.001). There was substantial heterogeneity in the measures of association across the studies (Figure 3) with an I^2^=91.6%, Q^2^=94.75, *P*<.001. The funnel plot (Multimedia Appendix 1) shows asymmetry, suggesting some publication bias being present; however, the Egger test for small-study effects was not significant (*P*=.30).

### Problematic Social Media Use and Stress

**Figure 4:**
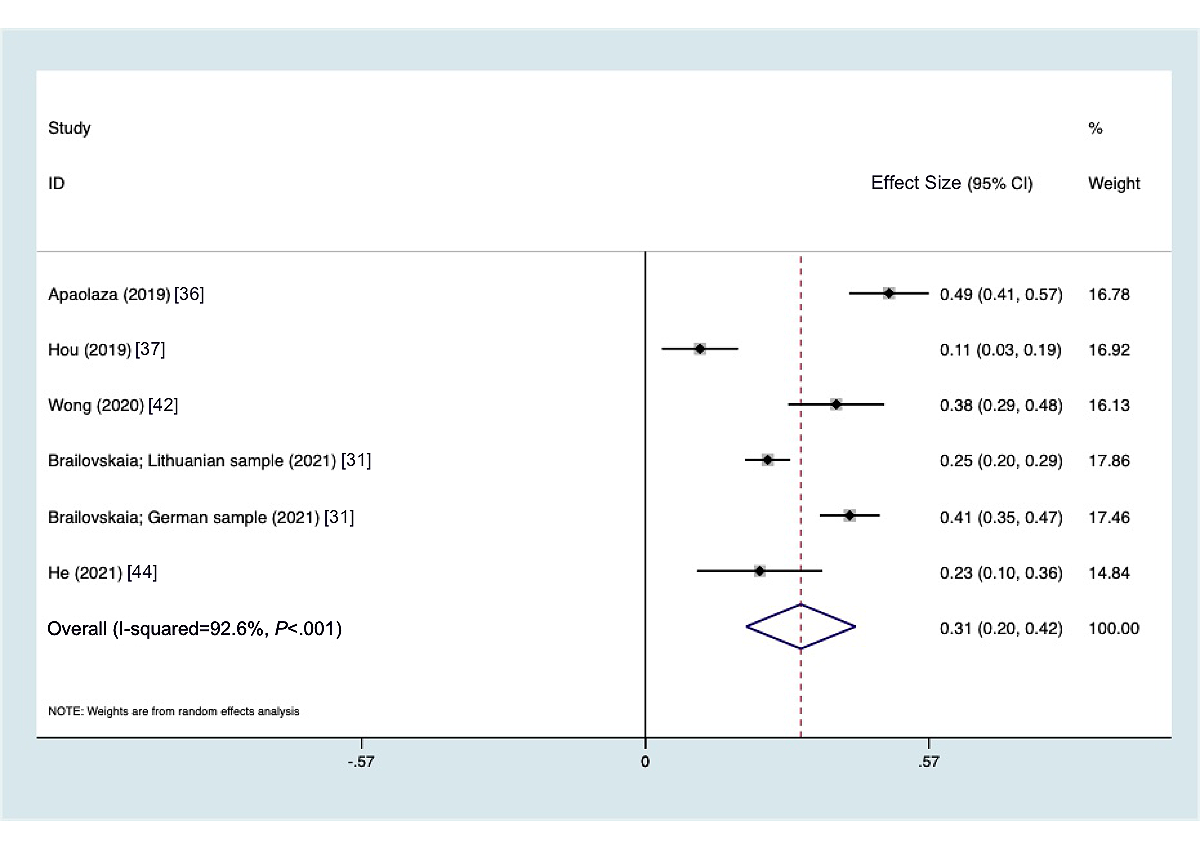
Forest plot of stress and problematic social media use by year.

Finally, when examining stress as an outcome, only 6 studies were identified measuring an association with problematic social media use in adolescents and young adults. The summary metaregression correlation between problematic social media use and stress was 0.313 (95% CI 0.203-0.423, *P*<.001). There was heterogeneity in the measures of association across the studies (Figure 4) with an I^2^=92.6%, Q^2^=67.59, *P*<.001. The funnel plot (Multimedia Appendix 1) shows symmetry, suggesting no publication bias, with no significant bias from the Egger test as well (*P*=.79).

### Moderators of Problematic Social Media Use

The metaregression assessing the impact of age as a covariate on the relationship between problematic social media use and all mental health outcomes combined showed that age was not significantly moderating the relationships (*P*=.75). When examining gender as a covariate in the relationship between problematic social media use and all mental health outcomes, gender did not significantly moderate the relationship (*P*=.25). Finally, year of publication also did not significantly moderate the relationship between problematic social media use and all mental health outcomes when added as a covariate (*P*=.09). See Multimedia Appendix 1 for metaregression plots.

## Discussion

### Principal Findings

This meta-analysis reports outcome measures of depression, anxiety, and stress in association with problematic social media use, specifically among adolescents and young adults. There is evidence for a significant relationship between problematic social media use in youth and negative mental health outcomes, particularly higher depression and anxiety symptoms, and greater stress. The strongest correlation was observed with anxiety; however, this also presented the most heterogeneity, likely due to the variety of assessments used to quantify symptoms of anxiety in the individual studies.

Although the correlations are moderate, this meta-analysis provides further evidence for the possible harms of problematic social media use. Previous meta-analyses examining time spent on social media and mental health show very small effect sizes, with most correlations being reported below *r*=0.20 [46-48]. One explanation for previously small correlations observed is the variability of social media content itself and the ways individuals are using or viewing their social media accounts. There has been evidence of multiple variables that can influence the severity of mental health outcomes such as night time–specific use, passive use, the number of social media platforms, motives for using social media, and so on [3,49-52]. Problematic social media use is a distinct pattern of use characterized by “addiction-like” symptoms based on behavioral and psychological attributes. It is characterized not only by time spent on social media, but also by measuring the extent of symptoms similar to a substance-related disorder, such as withdrawal, tolerance, and dependence [22]. Therefore, problematic social media use could represent a more clinically meaningful behavior to direct research, as a stronger relationship is seen with adverse mental health symptoms compared to previous studies investigating time spent on social networking sites or screen time in general [23,53,54].

The influence of age is still highly debated with evidence pointing toward younger social media users being more likely to have worse mental health symptoms compared to older users [55], whereas others have found no significant age effect with time spent on social media [56]. Cunningham et al [23] found age did not moderate the relationship between problematic social media use and depression; however, this study was performed in a mainly adult sample. Likewise, in our meta-analysis, age did not significantly moderate the relationship between the outcome variables combined and problematic use. This is likely due to the restricted age range, as the mean age between individual studies were analogous. Higher social media usage, along with developmental vulnerabilities, in adolescents and young adults has been proposed to explain the higher association with worse mental health outcomes compared to adults [57,58]. However, when looking specifically at mental health associated with problematic social media use as a behavior, the severity of reported problematic use symptoms may be more imperative to consider rather than age. Future research could directly compare adolescents to adults to examine if a difference in correlational strength is present, specifically when measuring problematic use.

Gender was examined as a moderator by including the percentage of male participants from each study into a metaregression analysis. Gender did not significantly moderate the relationship between problematic social media use and mental health, suggesting the association between mental health symptoms and problematic use of social media is not different between genders. Studies included in this meta-analysis did not specify if they assessed biological sex. Future research should provide more specific results for each group for both sex and genders to allow future meta-analyses to summarize this information and provide insight into gaps in the current literature on problematic use of social media [23,51,59].

Year of publication did not significantly moderate the relationship between problematic social media use and mental health outcomes. Although there are increased rates of social media use in adolescents and young adults over time, this may not be directly pertinent in the strength of the association between mental health and problematic use [23,60]. Year of publication may be more indicative of the prevalence of social media use as it increases with the growing use of technology [60]. Along with previous data, it is suggested that mental health symptoms associated with problematic social media use do not appear to be worsening over time; however, longitudinal studies exploring this specific aspect are needed.

### Strengths and Limitations

This study is not without limitations. The number of studies included in each meta-analysis was limited; therefore, the results are somewhat limited in power. Secondly, the results are based on cross-sectional correlational data. Therefore, a causal relationship cannot be inferred from the direct impact of social media on mental health outcomes of depressive symptoms, anxiety symptoms, or stress. It is possible that there are likely bidirectional effects between poor mental health and social media use [61]. In addition, the research studies included in the meta-analysis used did not report assessing the presence of a clinical diagnosis; therefore, it is unknown how many participants already had a known or possible clinical psychiatric diagnosis. This could influence the results of the outcome variables being measured, as it is unknown if individuals are more likely to have negative social media experiences or consequences as a result of using social media compared to individuals without a mental health diagnosis. Although the included questionnaires were previously validated, the majority of the research relies on self-report measures, also presenting as a limitation to the results reported.

### Future Directions

Overall, there is a lack of research providing evidence on the mental health outcomes of social media use, particularly patterns of problematic use in younger populations. In order to thoroughly understand the direct implications of problematic social media use, longitudinal studies could aid in providing more causational conclusions, as cross-sectional methodology is limited in its ability to draw conclusions beyond correlation [62]. In addition to a longitudinal design, understanding the biological basis of problematic use could contribute to understanding vulnerability to negative mental health outcomes. Future studies exploring the relationship between problematic social media and mental health outcomes would also benefit from including more detailed information on how participants are using various platforms. Indeed, there are several other scales exploring social media use that explore motivations for and mood associated with use (eg, social media use integration scale), which may provide greater depth of understanding around these associations. Finally, in traditional clinical practices for substance use disorders, treatment is often based on abstinence. For problematic social media use, total abstinence may not be a realistic option in today’s technology-based culture. Therefore, there should also be an increasing focus on identifying healthy ways to use social media in order to avoid the development of problematic use.

### Conclusions

The findings from this study provide further evidence of the association between problematic social media and negative mental health outcomes of depression, anxiety, and stress among adolescents and young adults. Although there is a large amount of evidence pointing toward the negative impacts of social media on mental health, there is still a need for further research to provide conclusive results on the causal relationship and how social media can be used without taking a toll on the mental health of users. Considering the omnipresence of social media among youth, more resources should be allocated to better understand the relationship between use and mental health symptoms and to prevent such negative outcomes.

## Appendix

Multimedia Appendix 1 Quality assessment rating for each study included in the meta-analysis. Questionnaires used for each study to measure problematic use and the outcome variables. Funnel plots and metaregressions.

## Abbreviations

PRISMA: Preferred Reporting Items for Systematic Reviews and Meta-Analyses
PROSPERO: Prospective Register of Systematic Reviews
