# Supplementary material for: Problematic Social Media Use in Adolescents and Young Adults: Systematic Review and Meta-analysis
Source: JMIR Ment Health. 2022 Apr 14;9(4):e33450. doi: 10.2196/33450 (PMC9052033; doi:10.2196/33450)
Supplement: Multimedia Appendix 1 [file mental_v9i4e33450_app1.docx]

Problematic Social Media Use in Adolescents and Young Adults: A Meta-Analysis

Appendix A

Table 1: Quality assessment rating for each study included in the meta-analysis.

| **First author (year)** | **Quality Rating** |
| --- | --- |
| Holmgren (2017) | 6 stars |
| Wang (2018) | 7 stars |
| Apaolaza (2019) | 5 stars |
| Hou (2019) | 6 stars |
| Kircaburun (2019) | 6 stars |
| Mitra (2019) | 5 stars |
| Kim (2020) | 5 stars |
| Kircaburun, Demetrovics (2020) | 7 stars |
| Kircaburun, Grifiths (2020) | 6 stars |
| Stockdale (2020) | 6 stars |
| Wong (2020) | 8 stars |
| Yildiz (2020) | 5 stars |
| Yunxiang (2020) | 7 stars |
| Brailovskaia; Lithuanian sample (2021) | 7 stars |
| Brailovskaia; German sample (2021) | 7 stars |
| Giordano (2021) | 7 stars |
| He (2021) | 6 stars |
| Kilincel (2021) | 7 stars |

Table 2: Questionnaires used for each study to measure problematic use and the outcome variables.

| First author (year) | Problematic Use Measure | Outcome Measure |
| --- | --- | --- |
| Holmgren (2017) | Problematic Use of Mobile Phone Scale (modified for social media use) | Center of Epidemiologic Studies Depression Scale |
| Wang (2018) | The Social Networking Intrusion Questionnaire | Center of Epidemiologic Studies Depression Scale |
| Apaolaza (2019) | Compulsive Mobile SNS Use | Perceived Stress Scale |
| Hou (2019) | Social Media Intrusion Questionnaire | Center of Epidemiologic Studies Depression Scale  State-Trait Anxiety Inventory  Perceived Stress Scale |
| Kircaburun (2019) | Bergen Social Media Addiction Scale | The Short Depression-Happiness Scale |
| Mitra (2019) | Social Media Disorder Scale | The Clinically Useful Depression Outcome Scale |
| Chen (2020) | Social Networking Websites Addiction Scale | Social Phobia Scale |
| Kim (2020) | Social Networking Addiction Tendency | Anxiety Tendency Questionnaire |
| Kircaburun, Demetrovics (2020) | Problematic Social Media Use Questionnaire | The Short Depression-Happiness Scale |
| Kircaburun, Grifiths (2020) | Problematic Social Media Use Questionnaire | The Short Depression-Happiness Scale |
| Stockdale (2020) | Problematic Use of Mobile Phone Scale (modified for social media use) | Center of Epidemiologic Studies Depression Scale  Spence Child Anxiety Inventory |
| Wong (2020) | Bergen Social Media Addiction Scale | The Depression Anxiety Stress Scale |
| Yildiz (2020) | Social Media Disorder Scale | Social Anxiety Scale for Adolescents |
| Brailovskaia; Lithuanian sample (2021) | Bergen Social Media Addiction Scale | The Depression Anxiety Stress Scale |
| Brailovskaia; German  sample (2021) | Bergen Social Media Addiction Scale | The Depression Anxiety Stress Scale |
| Giordano (2021) | Bergen Social Media Addiction Scale | Patient Health Questionnaire-4 |
| He (2021) | Social Media Dependence Questionnaire | Perceived Stress Scale |
| Kilincel (2021) | Social Media Disorder Scale | State-Trait Anxiety Inventory |


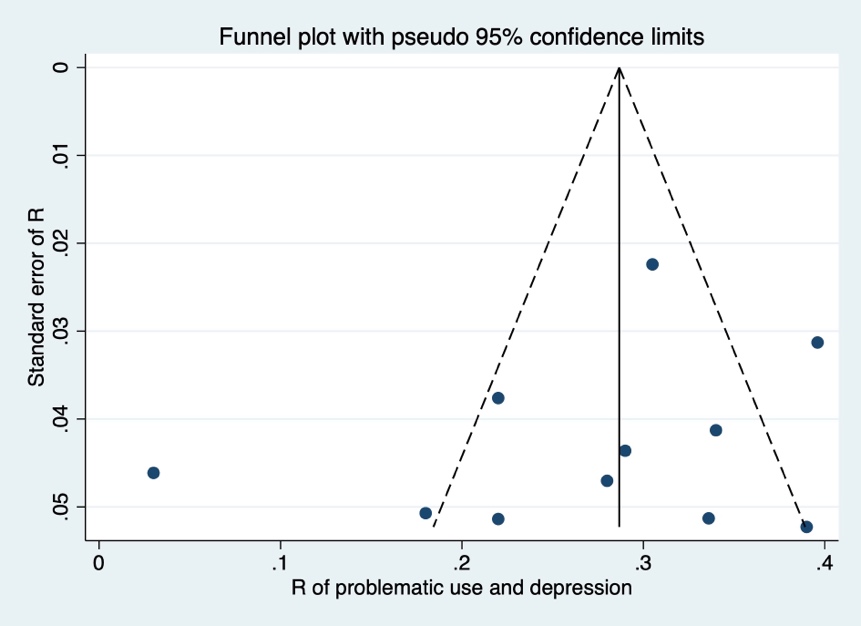


Figure 1. Funnel plot of depressive symptoms and problematic social media use.


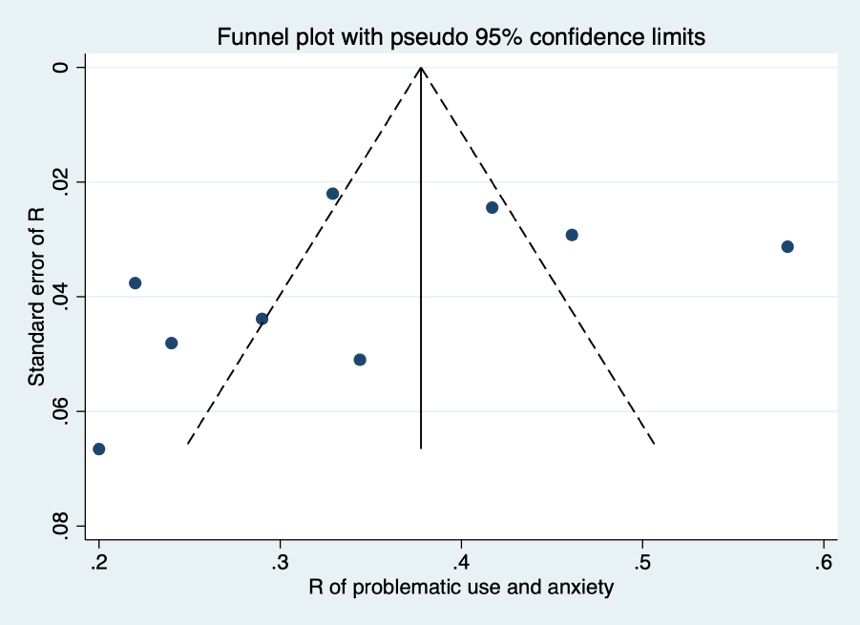


Figure 2. Funnel plot of anxiety symptoms and problematic social media use.


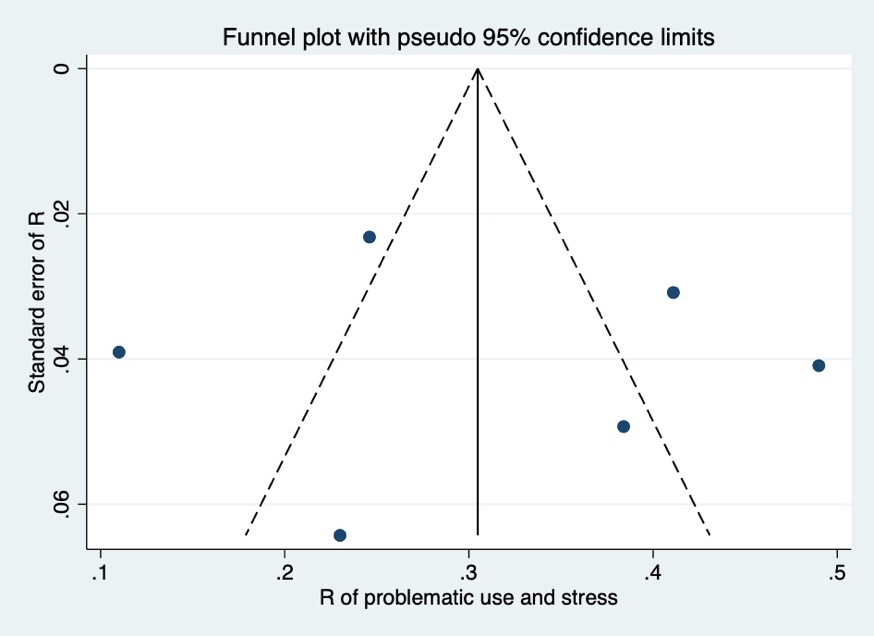


Figure 3. Funnel plot of stress and problematic social media use.


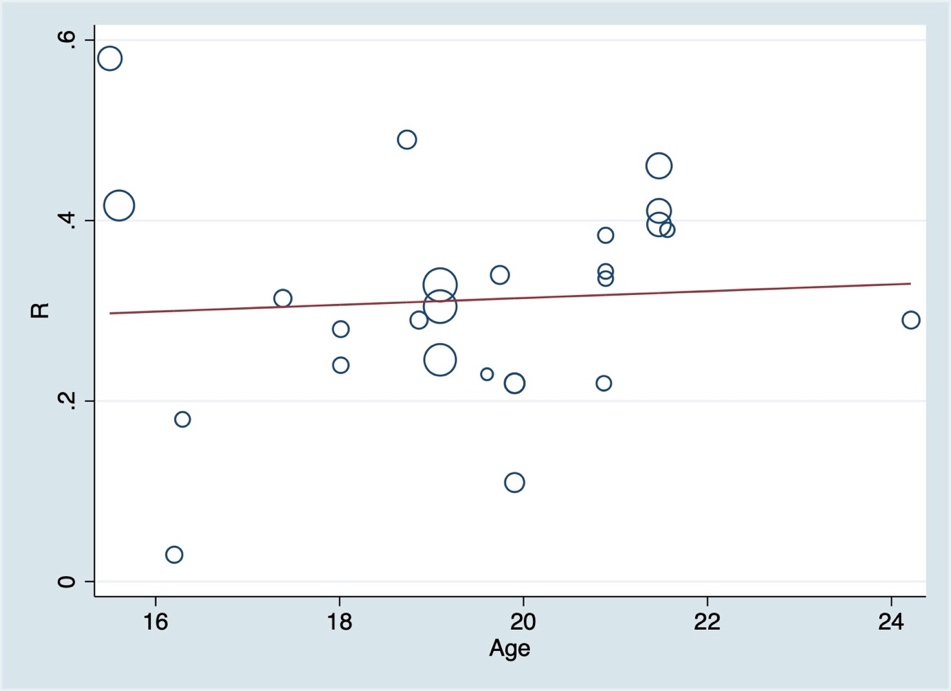


Figure 4. Meta-regression plot assessing age as a covariate


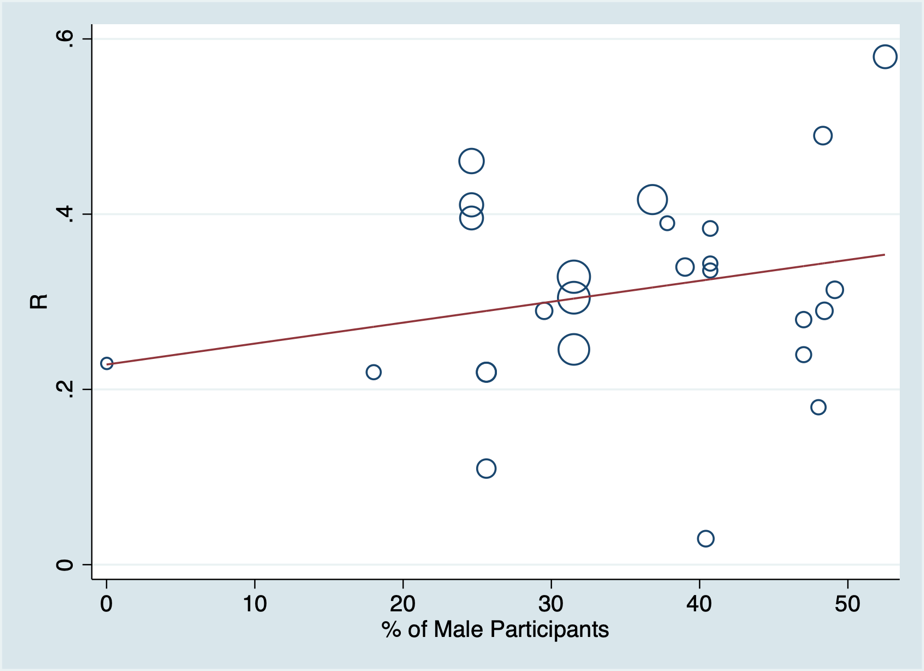


Figure 5. Meta-regression plot assessing sex as a covariate


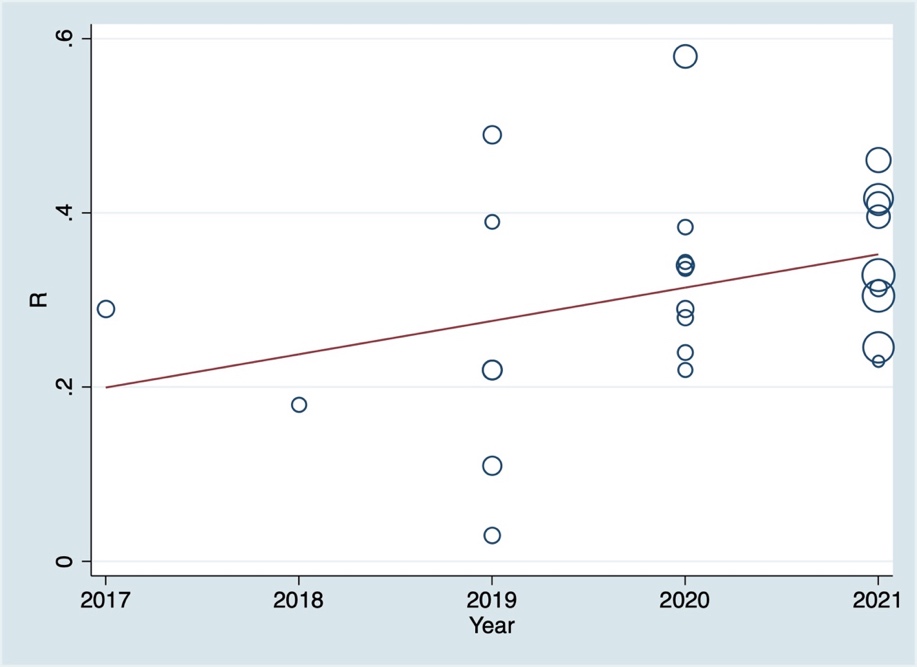


Figure 6. Meta-regression plot assessing year of publication as a covariate
